# Outcomes After Transcatheter Aortic Valve Replacement Among Medicare Beneficiaries: The Impact of Frailty and Social Vulnerability

**DOI:** 10.1016/j.shj.2025.100685

**Published:** 2025-06-24

**Authors:** Gregory P. Fontana, Harun Kundi, Steven V. Manoukian, Bruce Bowers, Todd M. Dewey, Charles T. Klodell, V. Seenu Reddy, John A. Riddick, Jorge A. Alvarez, Michael S. Chenier, Mark A. Groh, Marcos A. Nores, Pranav Loyalka, Francis J. Zidar, Julia B. Thompson, Maria C. Alu, David J. Cohen, Juan F. Granada, Martin B. Leon, Jeffrey J. Popma, Saibal Kar

**Affiliations:** a Los Robles Regional Medical Center, Thousand Oaks, California, USA; b HCA Healthcare, Nashville, Tennessee, USA; c Cardiovascular Research Foundation, New York, New York, USA; d Medical City Dallas, Dallas, Texas, USA; e HCA Florida North Florida Hospital, Gainesville, Florida, USA; f Tristar Centennial Medical Center, Nashville, Tennessee, USA; g Methodist Healthcare System, San Antonio, Texas, USA; h Mission Hospital, Asheville, North Carolina, USA; i HCA Florida JFK Hospital, Atlantis, Florida, USA; j HCA Houston Healthcare Medical Center, Houston, Texas, USA; k Heart Hospital of Austin, Austin, Texas, USA; l Columbia University Irving Medical Center/NewYork-Presbyterian Hospital, New York, New York, USA; m Beth Israel Deaconess Medical Center, Boston, Massachusetts, USA

**Keywords:** Frailty, Long-term mortality, Medicare, Social vulnerability, Transcatheter aortic valve replacement (TAVR)

## Abstract

**Background:**

Transcatheter aortic valve replacement (TAVR) is an accepted alternative to surgery in many patients with severe aortic stenosis. Clinical trials have evaluated early and late outcomes in selected TAVR patients, but predictors of late mortality have been less well studied in a broadly inclusive, national patient cohort undergoing TAVR. We sought to characterize 5-year outcomes after TAVR in Medicare beneficiaries and to evaluate the incremental predictive value of demographics, comorbidities, procedural factors, frailty, and social vulnerability in determining late mortality risk.

**Methods:**

We studied the fee-for-service Centers for Medicare & Medicaid Services MedPAR database that includes patients aged ≥65 years undergoing TAVR between 2017 and 2022. The primary endpoint was 5-year mortality. Sequential multivariable Cox models were constructed, incrementally adjusting for demographics, comorbidities, procedural and hospital characteristics, and frailty and social vulnerability. Model performance was assessed using C-statistics and integrated discrimination improvement (IDI).

**Results:**

A total of 371,248 TAVR patients were included in the analysis. The baseline model, including only demographic factors (age, sex, and race), yielded modest model performance (C = 0.589). Inclusion of comorbidities improved the model discrimination substantially (C = 0.684; IDI +6.9%, *p* < 0.001), and adding hospital and procedural characteristics yielded additional gains (C = 0.695; IDI +0.9%, *p* < 0.001). The final model integrated frailty and social vulnerability and achieved the highest predictive accuracy (C = 0.705; IDI +1.0%, *p* < 0.001).

**Conclusions:**

In this large national cohort, frailty and social vulnerability significantly improved risk prediction for long-term mortality after TAVR. We conclude that sociodemographic and frailty-related factors are important components for prediction of 5-year mortality after TAVR.

## Highlights

• We analyzed the 5-year outcomes of 371,248 patients who underwent transcatheter aortic valve replacement between 2017 and 2022 in the Center for Medicare & Medicaid Services Fee-for-Service MedPAR data. Patients were elderly (average age 80.3 years), frail (33.4%), and had a high percentage of increased social vulnerability (24.6%).
• In-hospital mortality was 1.14%, with the top six predictors including transapical access, admission with cardiogenic shock, emergent admission, urgent admission, high risk for frailty, and intermediate risk for frailty.
• The 5-year mortality rate was 52.1%, substantially lower than the age-sex-race-matched survival in the United States, with the highest predictive covariates being high frailty risk, metastatic tumor, liver disease, intermediate frailty risk, cancer, and admission for cardiogenic shock.
• After adjustment for age, sex, and race, incremental improvements of the predictive model were attained by adding comorbidities (by 6.9%), hospital and procedural characteristics (by 0.9%), and frailty and social vulnerability (1.0%).
• Our large-scale national study demonstrates that frailty and social vulnerability are independent predictors of long-term mortality following transcatheter aortic valve replacement and should be included in risk prediction models, particularly those serving high-risk and underserved populations.

Transcatheter aortic valve replacement (TAVR) is an accepted alternative to surgery in many patients with severe aortic stenosis. Randomized clinical studies have reported late clinical outcomes after TAVR in selected patients with varying surgical risk,^1^, ^2^, ^3^, ^4^, ^5^ yet late outcomes have been less well characterized in a broadly inclusive, national patient cohort undergoing TAVR, many of whom would not have qualified for clinical trials. With an increasing emphasis on TAVR outcome comparisons between hospitals,^6^^,^^7^ health care systems,^8^ and operators,^9^ precise risk adjustment models are needed to risk stratify late mortality, given that comorbidities in these patients often influence outcome well beyond their treated aortic valve disease.^10^ Prior studies have shown the importance of considering both traditional and nontraditional factors for intermediate-term mortality prediction in patients after TAVR^11^^,^^12^; however, the incremental utility of these factors in determining late risk remains understudied. The impact of social vulnerability has been studied in other populations, such as those undergoing joint replacement surgery^13^ and in the context of access to TAVR,^14^ but its association with TAVR outcomes has not been previously explored.

The objectives of our study were to characterize 5-year outcomes in a broadly inclusive, national cohort of older patients undergoing TAVR in the United States and to assess the incremental value of using traditional and nontraditional factors, including frailty and social vulnerability, in predictive models for long-term mortality.

## Methods

### Data Source

We utilized the Centers for Medicare & Medicaid Services (CMS) fee-for-service (FFS) MedPAR database to identify hospitalizations for TAVR between January 1, 2017, and December 31, 2022. The MedPAR file is an administrative claims database that includes 100% of inpatient claims for FFS Medicare beneficiaries, representing approximately 50% of Americans aged ≥65 years.^15^ TAVR cases were identified using International Classification of Diseases, 10th Revision, Procedure Coding System codes 02RF38H and 02RF38Z. To ensure consistent patient ascertainment, patients were included if they were continuously enrolled in FFS Medicare for at least 1 year prior to their index. For patients with multiple procedures within the study period, the first index procedure was selected. The study was determined by the Sterling Institutional Review Board to be exempt from institutional review board review under federal regulations, and informed consent was not required due to the retrospective nature of the research.

### Baseline Comorbidities

Baseline covariates were determined using secondary diagnosis codes marked as “present on admission” during the index hospitalization, supplemented with primary and secondary diagnosis codes from all hospitalizations occurring within 1 year prior to the index admission date for each patient (starting from January 1, 2016). The race/ethnicity of all beneficiaries was classified into three categories: White, Black, or other (including Asian, Hispanic, North American Native, other racial groups, and unknown). The Charlson Comorbidity Index was calculated for each patient to assess comorbid conditions.^16^^,^^17^

### Hospital and Procedural Characteristics

Hospital and procedural characteristics were assessed to examine differences in TAVR approaches, hospital resources, and admission types. The procedural approach (transapical vs. transfemoral) was identified using International Classification of Diseases, 10th Revision (ICD-10) procedure codes. The index procedure admission type (elective, urgent, or emergency) was determined from hospital admission records within the MedPAR database. Hospitals were categorized as teaching or nonteaching institutions based on CMS designations. TAVR procedural volume was calculated as the total number of TAVR procedures performed per hospital annually, and hospital capacity was assessed using the number of licensed hospital beds. These characteristics were analyzed to evaluate their relationship with patient outcomes and hospital-level variations in TAVR care.

### Frailty Assessment

Frailty was assessed using the Hospital Frailty Risk Score (HFRS), a validated tool based on administrative data developed in the United Kingdom.^18^ The HFRS was calculated for each individual patient using 109 ICD-10 diagnostic codes from all hospitalizations occurring within 1 year before the admission date of the primary hospitalization or from secondary diagnosis codes recorded as “present on admission” during the primary hospitalization (Supplemental Table 1). Patients were categorized into risk groups based on their HFRS, classified as low risk (<5), intermediate risk (5-15), or high risk (>15) for frailty, using previously established cutoff points. Those in the intermediate- and high-risk groups were considered frail.^18^

### Social Vulnerability Index

Patients' zip codes derived from Medicare files were first converted to county codes, which were then linked to data from the Centers for Disease Control and Prevention/Agency for Toxic Substances and Disease Registry to obtain Social Vulnerability Index (SVI) scores for each county.^13^ The 2020 dataset provided 4 summary theme ranking variables: socioeconomic status, household characteristics, racial and ethnic minority status, and housing type and transportation, along with the aggregate score for each theme to calculate an overall SVI score. Quartiles of the overall SVI score were used as the exposure variable, with Q1 (<0.25) representing the least vulnerable and Q4 (>0.75) representing the most vulnerable counties.

### Clinical Outcome Assessment

The primary outcome of the study was 5-year all-cause mortality, obtained from the Medicare Master Beneficiary Summary File. Time to death was calculated as the time between the date of the index procedure and the date of death. The secondary outcomes of the study were postprocedural stroke-, readmission-, and myocardial infarction-related rehospitalizations. Time to rehospitalization was calculated as the time period between the date of discharge from the index hospitalization and the date of admission for the first subsequent hospitalization. Patients were censored at the end of the follow-up period (December 31, 2022) or earlier if they were no longer enrolled in Medicare, as determined by the denominator file. Event rates for rehospitalizations were estimated using the Fine-Gray competing risks method, accounting for death as a competing event.

### Statistical Analysis

Continuous variables are presented as means and SDs or medians and interquartile ranges, and categorical variables are presented as counts and percentages. A variance inflation factor (VIF) analysis was performed to assess multicollinearity among predictors.^19^ Kaplan-Meier curves were constructed to visualize unadjusted 5-year mortality. Multivariable logistic regression analysis after adjustment of age, sex, race/ethnicity, comorbidities (each comorbidity was entered individually into the multivariable models rather than the Charlson Index), frailty, and social vulnerability-adjusted linear regression analyses was used to identify independent predictors of in-hospital mortality among patients who underwent TAVR. Multivariable Cox regression analysis was conducted to determine the independent predictors of 5-year all-cause long-term mortality with sequential adjustment for demographics, comorbidities, procedural/hospital factors, frailty, and SVI. All predictors, including frailty and SVI grades, were displayed as forest plots. Model performance was evaluated using C-statistics to measure overall discrimination, while the integrated discrimination improvement (IDI) method was used to quantify the incremental increase in discrimination across sequential risk assessment models.^20^ All statistical analyses were done in Stata MP, version 17.0 (StataCorp, College Station, TX) and SAS, version 9.4 (SAS Institute, Cary, NC) using a 2-tailed *p*-value of <0.05 to define statistical significance.

## Results

### Patient Demographics

A total of 371,248 patients who underwent TAVR between January 1, 2017, and December 31, 2022, were identified using specific procedure codes within CMS FFS MedPAR data. Yearly TAVR volumes among CMS FFS beneficiaries are shown in Table 1. Patient demographics are found in Table 2. The mean age was 80.3 years, and 45% were women. Frailty was present in 33.4% of patients, of whom approximately 1 in 5 had high frailty risk. SVI indicative of the highest quartile of vulnerability was present in 24.6% of patients. Transapical procedures were performed in 2.3% of patients, and 26.7% of patients were treated in teaching hospitals. TAVR procedures were emergent in 6.1% of patients and urgent in 8.6% of patients (Table 3).

**Table 1:** Yearly TAVR volume in CMS FFS patients

Year	Number (%)
2017	43,839 (11.8%)
2018	49,454 (13.3%)
2019	62,562 (16.9%)
2020	63,830 (17.2%)
2021	73,117 (19.7%)
2022	78,446 (21.1%)
Total	371,248 (100%)

Abbreviations: CMS, Center for Medicare & Medicaid Services; FFS, fee-for-service; TAVR, transcatheter aortic valve replacement.

**Table 2:** Baseline demographics in patients undergoing TAVR

Variables	Total
	N = 371,248
Age, y	80.3 ± 7.5
Sex, male	204,194 (55.0%)
Race/ethnicity	
White	340,829 (91.8%)
Black	14,232 (3.8%)
Other	16,187 (4.4%)
Myocardial infarction	49,987 (13.5%)
Congestive heart failure	275,656 (74.3%)
Peripheral vascular disease	85,327 (23.0%)
Cerebrovascular disease	45,428 (12.2%)
Dementia	20,298 (5.5%)
Chronic pulmonary disease	105,741 (28.5%)
Connective tissue disease	18,314 (4.9%)
Peptic ulcer disease	6973 (1.9%)
Diabetes without complications	93,007 (25.1%)
Diabetes with complications	90,382 (24.3%)
Hemiplegia or paraplegia	3426 (0.9%)
Renal disease	136,452 (36.8%)
Cancer (any malignancy)	23,494 (6.3%)
Liver disease (≥moderate)	3583 (1.0%)
Metastatic solid tumor	3554 (1.0%)
AIDS/HIV	230 (0.1%)

*Notes.* Continuous variables are expressed as mean ± SD; ordinal variables are expressed as frequencies, N (%).

Abbreviation: TAVR, transcatheter aortic valve replacement.

**Table 3:** Frailty, social vulnerability, and procedural details in patients undergoing TAVR

Variables	N = 371,248
Frailty groups	
Low-risk	247,490 (66.7%)
Intermediate-risk	97,130 (26.2%)
High-risk	26,628 (7.2%)
Frail	123,758 (33.4)
Quartiles of SVI	
1 (least vulnerable)	93,368 (25.1%)
2	93,146 (25.1%)
3	93,304 (25.1%)
4 (most vulnerable)	91,430 (24.6%)
Procedural and hospital characteristics	
Transapical approach	8692 (2.3%)
Type of admission	
Elective	316,804 (85.3%)
Urgent	31,841 (8.6%)
Emergent	22,603 (6.1%)
Teaching hospital	99,247 (26.7%)
TAVR volume over study period	317 [120-573]
Number of hospital beds	409 [293-597]

*Notes.* Continuous variables are expressed as median [interquartile range]; ordinal variables are expressed as frequencies, N (%).

Abbreviations: SVI, Social Vulnerability Index; TAVR, transcatheter aortic valve replacement.

### Predictors of In-Hospital Mortality

**Figure 1:**
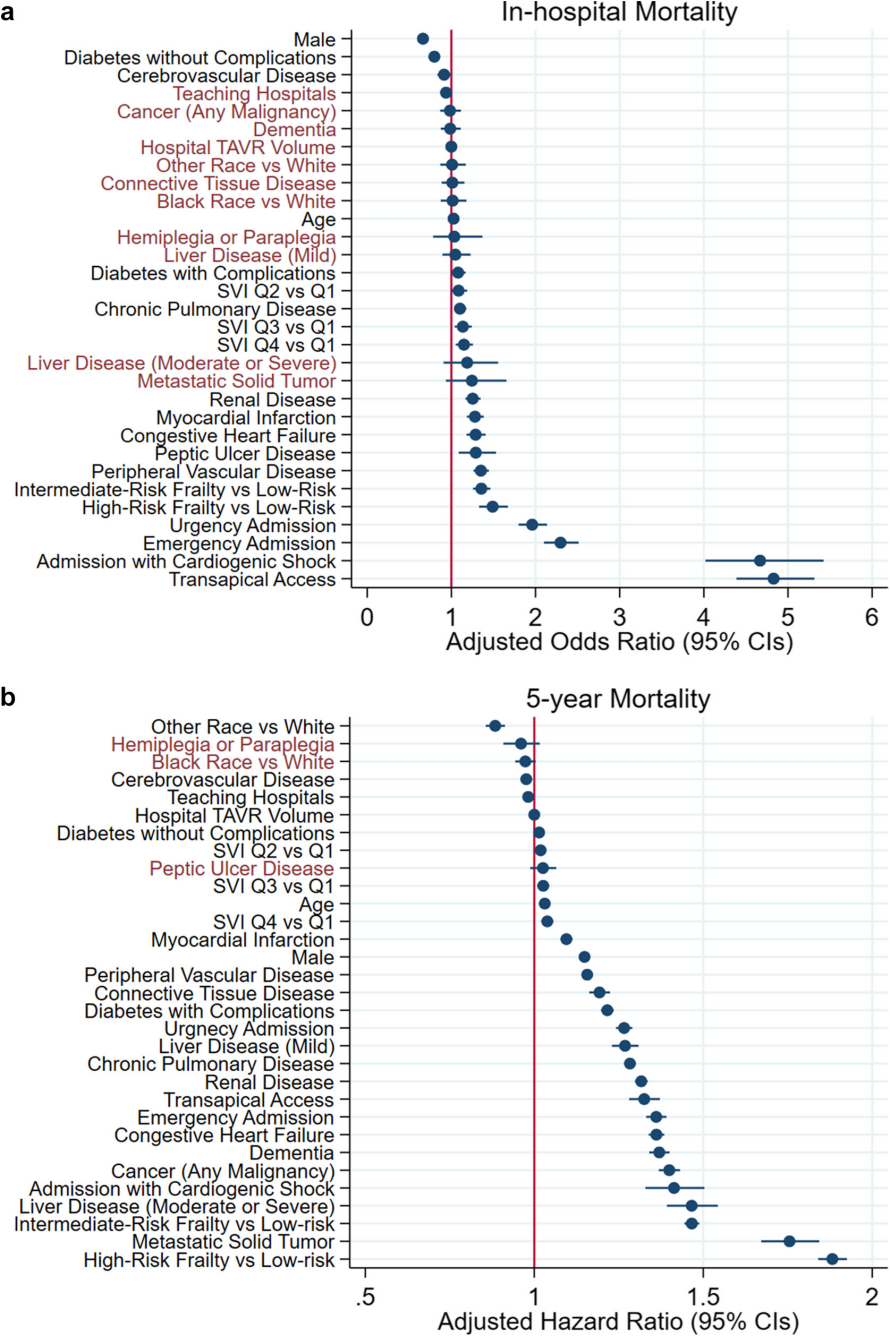
Multivariable predictors of in-hospital (a) and 5-year (b) mortality. Factors listed in black are significant predictors, whereas factors listed in red are not significant predictors. Reference for SVI is Q1 (least vulnerable), for frailty is low-risk, and for admission type is elective TAVR. Abbreviations: SVI, Social Vulnerability Index; TAVR, transcatheter aortic valve replacement.

**Figure 2:**
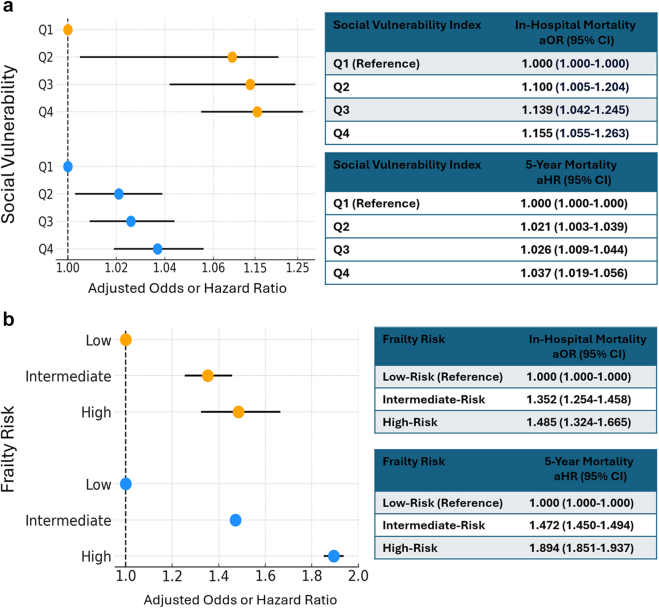
**Impact of frailty and SVI on in-hospital and 5-year all-cause mortality.** The forest plot shows the impact of the SVI and Frailty classification on in-hospital and 5-year mortality. Quartile 1 was the lowest risk for social vulnerability, and quartile 4 was the highest risk for social vulnerability. Intermediate and high frailty risk were compared to low risk for frailty. The upper panel indicates SVI and the bottom panel illustrates Frailty. Abbreviation: SVI, Social Vulnerability Index.

The observed in-hospital mortality rate was 1.14% (Table 4). The top six predictors for in-hospital mortality were transapical access, admission with cardiogenic shock, emergency admission, urgent admission, high risk for frailty, and intermediate risk for frailty (Figure 1a). The impact of SVI and frailty on in-hospital mortality is shown in Figure 2. A significant incremental increase in in-hospital mortality was observed across SVI quartiles and frailty risk levels. Compared to patients in the lowest quartile of SVI (Q1), those in the highest quartile (Q4) had significantly higher in-hospital mortality, with an adjusted odds ratio of 1.155 (95% CI: 1.055-1.263; *p* = 0.002). High frailty risk was also independently associated with increased in-hospital mortality (adjusted odds ratio: 1.485; 95% CI: 1.324-1.665).

**Table 4:** Unadjusted clinical event rates after TAVR

Outcomes	Rates, % (95% CI)
Mortality rates	
In-hospital	1.14% (1.11-1.17)
1-y∗	11.4% (11.2-11.4)
2-y∗	21.0% (20.9-21.2)
3-y∗	31.2% (31.0-31.4)
4-y∗	41.9% (41.7-42.2)
5-y∗	52.1% (51.8-52.4)
Rehospitalization rates	
Stroke∗	
In-hospital	
1-y	3.1% (2.9-3.4)
2-y	4.9% (4.4-5.1)
3-y	6.5% (5.9-6.8)
4-y	7.8% (7.3-8.4)
5-y	9.0% (8.8-9.1)
Heart failure†	
1-y	28.2% (28.0-28.3)
2-y	38.1% (37.9-38.3)
3-y	45.5% (45.3-45.6)
4-y	51.6% (51.3-51.8)
5-y	56.7% (56.4-57.0)
Acute myocardial infarction‡	
1-y	4.9% (4.8-5.0)
2-y	8.0% (7.9-8.1)
3-y	10.7% (10.6-10.8)
4-y	13.2% (13.1-13.4)
5-y	15.5% (15.3-15.7)

Abbreviation: TAVR, transcatheter aortic valve replacement.

∗ I63 and G45, at subsequent index.

† I5021, I5031, I5041, I5023, I5033, and I5043 at subsequent index.

‡ I21 at subsequent index.

### Predictors of 5-Year Mortality

**Figure 3:**
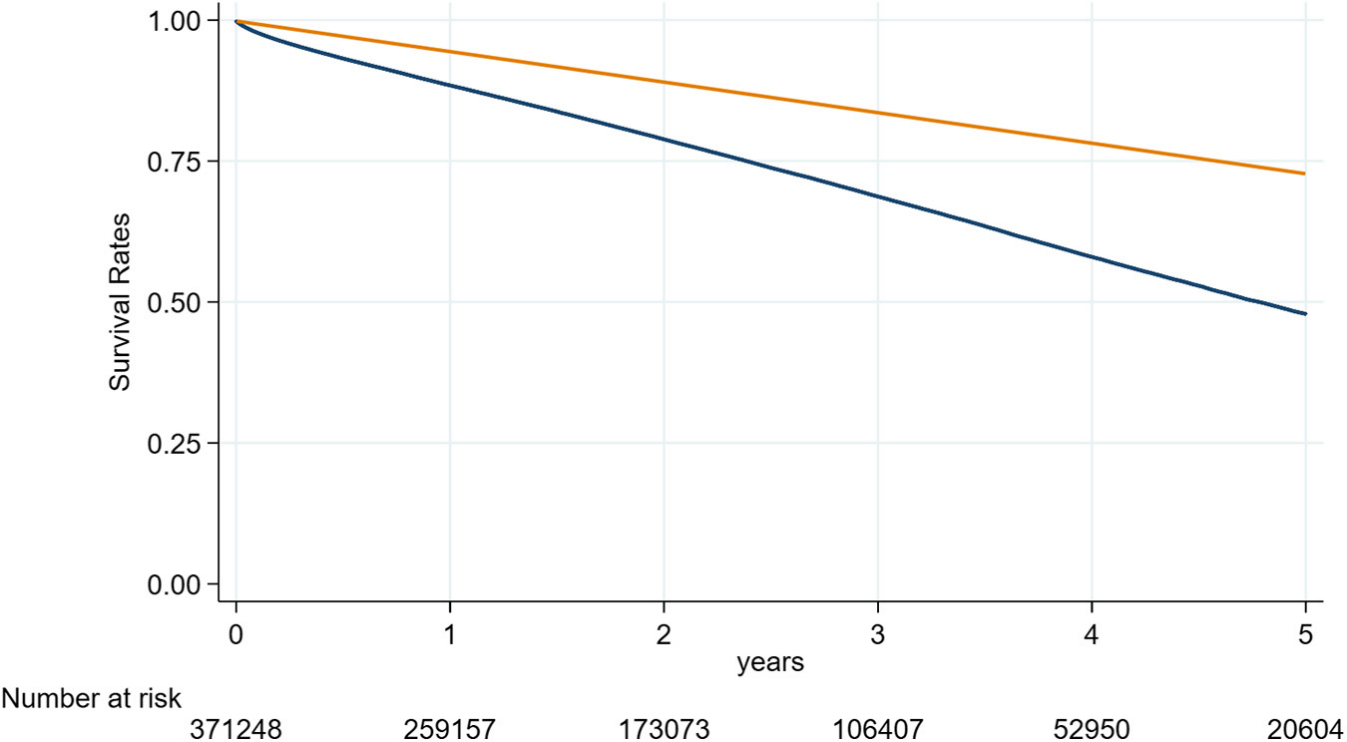
**Five-year survival by TAVR vs. age-sex-race-matched US life expectancy.** Unadjusted mortality rates in blue (TAVR). The orange line indicates the age-sex-race-matched US survival curves from life tables. Source for life tables: https://www.cdc.gov/nchs/data/nvsr/nvsr72/nvsr72-12.pdf. Abbreviation: TAVR, transcatheter aortic valve replacement.

Yearly event rates are found in Table 4, with mortality rates of 11.4% (11.2-11.4) at 1 year, 21.0% (20.9-21.2) at 2 years, 31.2% (31.0-31.4) at 3 years, 41.9% (41.7-42.2) at 4 years, and 52.1% (51.8-52.4) at 5 years. The 5-year Kaplan-Meier survival curve plotted against age-sex-race-matched US life expectancy is shown in Figure 3. The six most predictive covariates for 5-year mortality were high frailty risk, metastatic tumor, liver disease, intermediate frailty risk, cancer, and admission for cardiogenic shock (Figure 1b). The impact of both SVI and frailty on 5-year mortality is shown in Figure 2b. Five-year all-cause mortality gradually increased with higher levels of SVI and frailty. Five-year all-cause mortality was significantly higher among patients in the highest SVI quartile (Q4) compared to the lowest (Q1), with an adjusted hazard ratio of 1.037 (95% CI: 1.019-1.056; *p* < 0.001). Frailty was also a strong predictor of 5-year all-cause mortality; patients with high frailty risk had an adjusted hazard ratio of 1.894 (95% CI: 1.851-1.937; *p* < 0.001).

### Incremental Risk-Adjusted Mortality Models

Incremental adjustment for the contributors to 5-year mortality risk is found in Table 5. Model 1 was adjusted only for age, sex, and race; the C-statistic was 0.589 (95% CI: 0.587-0.592). Model 2 included the covariates from model 1 with the addition of comorbidities; the C-statistic improved to 0.684 (95% CI: 0.682-0.685), and the IDI improved by 6.9% (*p* < 0.001). Model 3 used model 2 and added hospital and procedural characteristics; the C-statistic improved to 0.695 (95% CI: 0.693-0.697), and the IDI improved by 0.9% (*p* < 0.001). Model 4 included model 3 with the addition of frailty and social vulnerability; the C-statistic increased to 0.705 (95% CI: 0.703-0.706), and the IDI improved by 1.0% (*p* < 0.001). VIF analysis indicated minimal collinearity, with a mean VIF of 1.12 and all individual VIFs below 1.5.

**Table 5:** Discrimination improvement across Cox models for predicting 5-year mortality

Models	C-statistic (95% CIs)	IDI (%)	IDI *p*-value	DeLong *p*-value
Model 1 (adjusted for age, sex, and race)	0.589 (0.587-0.592)	-	-	-
Model 2 (model 1 + comorbidities)	0.684 (0.682-0.685)	6.9%∗	<0.001∗	<0.001∗
Model 3 (model 2 + hospital and procedural characteristics)	0.695 (0.693-0.697)	0.9%†	<0.001†	<0.001†
Model 4 (model 3 + SVI and frailty)	0.705 (0.703-0.706)	1.0%‡	<0.001‡	<0.001

Abbreviations: IDI, integrated discrimination improvement; SVI, Social Vulnerability Index.

∗ Model 2 vs. model 1.

† Model 3 vs. model 2.

‡ Model 4 vs. model 3

### Additional 5-Year Endpoints

The yearly frequency of additional clinical endpoints is found in Table 4. Kaplan-Meier curves illustrating the cumulative incidence of stroke, heart failure rehospitalization, and postprocedural acute myocardial infarction are presented in Supplemental Figure 1a-c, respectively. Following TAVR, the cumulative incidence of stroke reached 3.1% at 1 year and 9.0% at 5 years. Heart failure rehospitalizations were more frequent, with cumulative rates of 28.2% at 1 year and 56.7% at 5 years. The cumulative incidence of acute myocardial infarction was 4.9% at 1 year and increased to 15.5% at 5 years. Cumulative event rates were estimated using Fine-Gray competing risk methodology, treating death as a competing risk.

## Discussion

In this national cohort study of more than 370,000 FFS Medicare beneficiaries undergoing TAVR, representing approximately 50% of TAVR patients aged 65 years and over in the United States,^15^ both frailty and social vulnerability emerged as independent predictors of 5-year mortality. These findings emphasize the importance of comprehensive risk adjustment in evaluating long-term outcomes. We found the discriminative capacity of our models improved substantially with each successive layer of adjustment. The C-statistics increased from 0.589 with demographic variables alone to 0.705 in the fully adjusted model. The addition of comorbidities resulted in the most substantial improvement in model performance; however, further gains were observed with the inclusion of frailty and, notably, social vulnerability. Improvements in discrimination were corroborated by IDI analysis. Together, these results support incorporating both clinical and nontraditional risk factors such as frailty and social vulnerability into contemporary risk models for older adults undergoing TAVR.

### Mortality After TAVR

We report an in-hospital mortality rate of 1.14% in a broadly inclusive, national cohort of patients undergoing TAVR with an average age of 80 years. These results compare favorably with the results from the randomized clinical trial outcomes in patients treated with TAVR,^21^, ^22^, ^23^, ^24^ likely due to better patient selection and improved operator technique and transvalvular design. We found that transapical access, admission with cardiogenic shock, urgent or emergent admissions, and frailty risk, among other factors, increased in-hospital mortality, whereas teaching hospital status, hospital TAVR volume, liver disease, cancer, and Black compared with White race did not.

Five-year mortality rates in the randomized TAVR vs. surgery trials are associated with surgical risk: 5-year mortality was 10% to 13.5% in low-surgical-risk patients^1^^,^^2^; 30% to 46% in intermediate-surgical-risk^3^^,^^25^; and 55.3% to 67.8% in high-surgical-risk patients.^4^^,^^5^ We used the Medicare Master Beneficiary Summary File to identify deaths in patients undergoing TAVR; the Summary File is considered highly accurate for the detection of deaths in the FFS CMS database.^26^ Others have used linkage with the National Death Index to report TAVR late mortality modeled by surgical risk with lower reported mortality rates,^27^ although there is some loss of information with National Death Index depending on the quality of the linkage.^28^^,^^29^

We report a 5-year mortality rate of 52.5% in patients aged 65 years and older undergoing TAVR, with the highest risk in patients with frailty, metastatic cancer, and liver disease, among other factors. These findings suggest that late mortality after TAVR is driven by factors not related to the correction of aortic stenosis but instead due to underlying clinical and social factors that drive nonaortic valve mortality. Moreover, we constructed mortality predictive models that show independent incremental discrimination with the inclusion of comorbidities, hospital and procedural considerations, and frailty and social vulnerability.

### Frailty Assessment

Frailty is a well-established determinant of adverse outcomes in patients with cardiovascular disease, including TAVR.^10^, ^11^, ^12^^,^^30^, ^31^, ^32^, ^33^, ^34^, ^35^ In our analysis, high frailty risk was associated with an 89% increase in 5-year mortality relative to patients with low frailty risk. Intermediate frailty conferred a 47% higher mortality risk. Our findings further confirm the utility of the Hospital Frailty Risk Score, a claims-based tool derived solely from ICD-10 codes, as a robust instrument for predicting long-term mortality in the TAVR population. These findings reinforce the prognostic value of frailty assessment in preprocedural risk stratification for patients undergoing TAVR.

### Social Vulnerability

While it is well recognized that social disadvantage has important health care implications,^13^^,^^36^^,^^37^ social vulnerability has not been well studied in patients treated for valvular heart disease. The area deprivation index, an index that includes factors such as socioeconomic status, household characteristics, racial and ethnic minority status, and housing type and transportation, was used to show a higher 30-day mortality among beneficiaries residing in the most disadvantaged neighborhoods.^38^ To our knowledge, this study represents the first national analysis linking area-level social vulnerability to long-term mortality following TAVR. After adjustment for clinical risk, patients residing in the most socially vulnerable counties continued to exhibit higher in-hospital and 5-year mortality than those from less vulnerable areas. These results suggest that community-level disadvantages may independently influence postprocedural outcomes and support the potential utility of SVI in efforts to address health care disparities and inform value-based reimbursement strategies.

## Limitations

There are several important limitations of this analysis. First, the CMS FFS claims data excludes a substantial proportion of the U.S. older adult population, including the approximately 50% of Medicare beneficiaries aged 65 years and older who are enrolled in Medicare Advantage plans.^15^ Accordingly, our analysis does not include patients under 65 years old. Additionally, patients who receive care through integrated or federal health systems—such as Kaiser Permanente or the Veterans Health Administration—are not captured in FFS claims data, limiting the generalizability of these findings to the broader Medicare population. Nevertheless, outcomes and predictors should be similar in these populations. Second, claims data do not contain important information about system or operator status, echocardiographic severity, or anatomic morphology of the aortic annulus, limiting further classification of the severity of the aortic stenosis. Finally, our 5-year mortality rates are higher than reported in other studies, but these studies restricted their analysis by age,^27^ low surgical risk,^39^ and presence of coronary artery disease requiring revascularization.^40^

## Conclusions

This large-scale national study demonstrates that frailty and social vulnerability are independent predictors of long-term mortality following TAVR. These findings advocate for the incorporation of comprehensive risk adjustment, including demographic, clinical, and social factors, when evaluating outcomes, particularly those serving high-risk and underserved populations.

## Ethics Statement

This study was carried out in accordance with all appropriate ethical guidelines. It was approved under a waiver by Sterling Institutional Review Board (IRB). The research involved analysis of Medicare Research Identifiable Files (RIFs), which contain protected health information and require appropriate data use agreements. The study was determined to be exempt from IRB review under federal regulations, and informed consent was not required due to the retrospective nature of the research.

## Funding

This study was supported, in part, by 10.13039/100016646HCA Healthcare.

## Disclosure Statement

G. P. Fontana reports grant support from Abbott Vascular, Medtronic, Edwards Lifesciences, V-Wave, 4C Medical, Pi-Cardia, Emblock, and CroiValve; consulting fees from Abbott Vascular, Medtronic, and JenaValve; and steering committee participation with Janssen Pharmaceuticals. F. Zidar reports consultant fees from Edwards Lifesciences, Medtronic, and Abbott. D. J. Cohen reports institutional research support from Abbott, Edwards Lifesciences, Boston Scientific, Philips, Corvia Medical, Brain-Q, Saranas, Zoll Medical, CathWorks, and ANCORA and consulting income from Medtronic, Edwards Lifesciences, Abbott, Boston Scientific, Corvia Medical, Impulse Dynamics, AngioInsight, and HeartBeam. J. F. Granada is co-founder of Cephea Valve Technologies (Abbott) and CEO of the Cardiovascular Research Foundation. M. B. Leon reports institutional research support from Edwards Lifesciences, Medtronic, Boston Scientific, and Abbott, and consulting/advisory board participation for Foldax, Anteris, JenaValve, Medinol, SoloPace, and Bain Capital. J. J. Popma is a former employee of Medtronic and reports nonvested equity in Medtronic. S. Kar reports consulting fees/advisory board participation with Boston Scientific, consulting fees/stock equity with Valcare, and consulting fees from Medtronic. The other authors had no conflicts to declare.

## Review Statement

The review of this manuscript was managed by Guest Editor Harry Dauerman, MD.
