# Supplementary material for: Outcomes After Transcatheter Aortic Valve Replacement Among Medicare Beneficiaries: The Impact of Frailty and Social Vulnerability
Source: Struct Heart. 2025 Jun 24;9(8):100685. doi: 10.1016/j.shj.2025.100685 (PMC12399245; doi:10.1016/j.shj.2025.100685)
Supplement: Supplementary Materials [file mmc1.docx]

**Outcomes After TAVR Among Medicare Beneficiaries: The Impact of Frailty and Social Vulnerability (G.P. Fontana, et al.)**

**SUPPLEMENTAL APPENDIX**

**Supplementary Table S1. ICD-10 Codes Used for the “Hospital Frailty Risk” Score**

|  |  | **Point** |
| --- | --- | --- |
| **G81** | Hemiplegia | 4·4 |
| **G30** | Alzheimer's disease | 4·0 |
| **I69** | Sequelae of cerebrovascular disease (secondary codes) | 3·7 |
| **R29** | Other symptoms and signs involving the nervous and musculoskeletal systems (R29·6 Tendency to fall) | 3·6 |
| **N39** | Other disorders of urinary system (includes urinary tract infection and urinary incontinence) | 3·2 |
| **F05** | Delirium, not induced by alcohol and other psychoactive substances | 3·2 |
| **W19** | Unspecified fall | 3·2 |
| **S00** | Superficial injury of head | 3·2 |
| **R31** | Unspecified hematuria | 3·0 |
| **B96** | Other bacterial agents as the cause of diseases classified to other chapters (secondary code) | 2·9 |
| **R41** | Other symptoms and signs involving cognitive functions and awareness | 2·7 |
| **R26** | Abnormalities of gait and mobility | 2·6 |
| **I67** | Other cerebrovascular diseases | 2·6 |
| **R56** | Convulsions, not elsewhere classified | 2·6 |
| **R40** | Somnolence, stupor and coma | 2·5 |
| **T83** | Complications of genitourinary prosthetic devices, implants and grafts | 2·4 |
| **S06** | Intracranial injury | 2·4 |
| **S42** | Fracture of shoulder and upper arm | 2·3 |
| **E87** | Other disorders of fluid, electrolyte and acid-base balance | 2·3 |
| **M25** | Other joint disorders, not elsewhere classified | 2·3 |
| **E86** | Volume depletion | 2·3 |
| **R54** | Senility | 2·2 |
| **F03** | Unspecified dementia | 2·1 |
| **W18** | Other fall on same level | 2·1 |
| **Z75** | Problems related to medical facilities and other health care | 2·0 |
| **F01** | Vascular dementia | 2·0 |
| **S80** | Superficial injury of lower leg | 2·0 |
| **L03** | Cellulitis | 2·0 |
| **H54** | Blindness and low vision | 1·9 |
| **E53** | Deficiency of other B group vitamins | 1·9 |
| **Z60** | Problems related to social environment | 1·8 |
| **G20** | Parkinson's disease | 1·8 |
| **R55** | Syncope and collapse | 1·8 |
| **S22** | Fracture of rib(s), sternum and thoracic spine | 1·8 |
| **K59** | Other functional intestinal disorders | 1·8 |
| **N17** | Acute renal failure | 1·8 |
| **L89** | Decubitus ulcer | 1·7 |
| **Z22** | Carrier of infectious disease | 1·7 |
| **B95** | Streptococcus and staphylococcus as the cause of diseases classified to other chapters | 1·7 |
| **L97** | Ulcer of lower limb, not elsewhere classified | 1.6 |
| **R44** | Other symptoms and signs involving general sensations and perceptions | 1·6 |
| **K26** | Duodenal ulcer | 1·6 |
| **I95** | Hypotension | 1·6 |
| **N19** | Unspecified renal failure | 1·6 |
| **A41** | Other septicemia | 1·6 |
| **Z87** | Personal history of other diseases and conditions | 1·5 |
| **J96** | Respiratory failure, not elsewhere classified | 1·5 |
| **M19** | Other arthrosis | 1·5 |
| **G40** | Epilepsy | 1·5 |
| **M81** | Osteoporosis without pathological fracture | 1·4 |
| **S72** | Fracture of femur | 1·4 |
| **S32** | Fracture of lumbar spine and pelvis | 1·4 |
| **E16** | Other disorders of pancreatic internal secretion | 1·4 |
| **R94** | Abnormal results of function studies | 1·4 |
| **N18** | Chronic renal failure | 1·4 |
| **R33** | Retention of urine | 1·3 |
| **R69** | Unknown and unspecified causes of morbidity | 1·3 |
| **N28** | Other disorders of kidney and ureter, not elsewhere classified | 1·3 |
| **R32** | Unspecified urinary incontinence | 1·2 |
| **G31** | Other degenerative diseases of nervous system, not elsewhere classified | 1·2 |
| **Y95** | Nosocomial condition | 1·2 |
| **S09** | Other and unspecified injuries of head | 1·2 |
| **R45** | Symptoms and signs involving emotional state | 1·2 |
| **G45** | Transient cerebral ischemic attacks and related syndromes | 1·2 |
| **Z74** | Problems related to care-provider dependency | 1·1 |
| **M79** | Other soft tissue disorders, not elsewhere classified | 1·1 |
| **W06** | Fall involving bed | 1.1 |
| **S01** | Open wound of head | 1.1 |
| **A04** | Other bacterial intestinal infections | 1·1 |
| **A09** | Diarrhea and gastroenteritis of presumed infectious origin | 1.1 |
| **J18** | Pneumonia, organism unspecified | 1·1 |
| **J69** | Pneumonitis due to solids and liquids | 1·0 |
| **R47** | Speech disturbances, not elsewhere classified | 1·0 |
| **E55** | Vitamin D deficiency | 1·0 |
| **Z93** | Artificial opening status | 1·0 |
| **R63** | Symptoms and signs concerning food and fluid intake | 0·9 |
| **H91** | Other hearing loss | 0·9 |
| **W10** | Fall on and from stairs and steps | 0·9 |
| **W01** | Fall on same level from slipping, tripping and stumbling | 0·9 |
| **E05** | Thyrotoxicosis [hyperthyroidism] | 0·9 |
| **M41** | Scoliosis | 0·9 |
| **R13** | Dysphagia | 0·8 |
| **Z99** | Dependence on enabling machines and devices | 0·8 |
| **M80** | Osteoporosis with pathological fracture | 0·8 |
| **K92** | Other diseases of digestive system | 0·8 |
| **I63** | Cerebral Infarction | 0·8 |
| **N20** | Calculus of kidney and ureter | 0·7 |
| **F10** | Mental and behavioral disorders due to use of alcohol | 0·7 |
| **Y84** | Other medical procedures as the cause of abnormal reaction of the patient | 0·7 |
| **R00** | Abnormalities of heartbeat | 0·7 |
| **J22** | Unspecified acute lower respiratory infection | 0·7 |
| **Z73** | Problems related to life-management difficulty | 0·6 |
| **R79** | Other abnormal findings of blood chemistry | 0·6 |
| **Z91** | Personal history of risk-factors, not elsewhere classified | 0·5 |
| **S51** | Open wound of forearm | 0·5 |
| **F32** | Depressive episode | 0·5 |
| **M48** | Spinal stenosis (secondary code only) | 0·5 |
| **E83** | Disorders of mineral metabolism | 0·4 |
| **M15** | Polyarthritis | 0·4 |
| **D64** | Other anemias | 0·4 |
| **L08** | Other local infections of skin and subcutaneous tissue | 0·4 |
| **R11** | Nausea and vomiting | 0·3 |
| **K52** | Other noninfective gastroenteritis and colitis | 0·3 |
| **R50** | Fever of unknown origin | 0·1 |

**
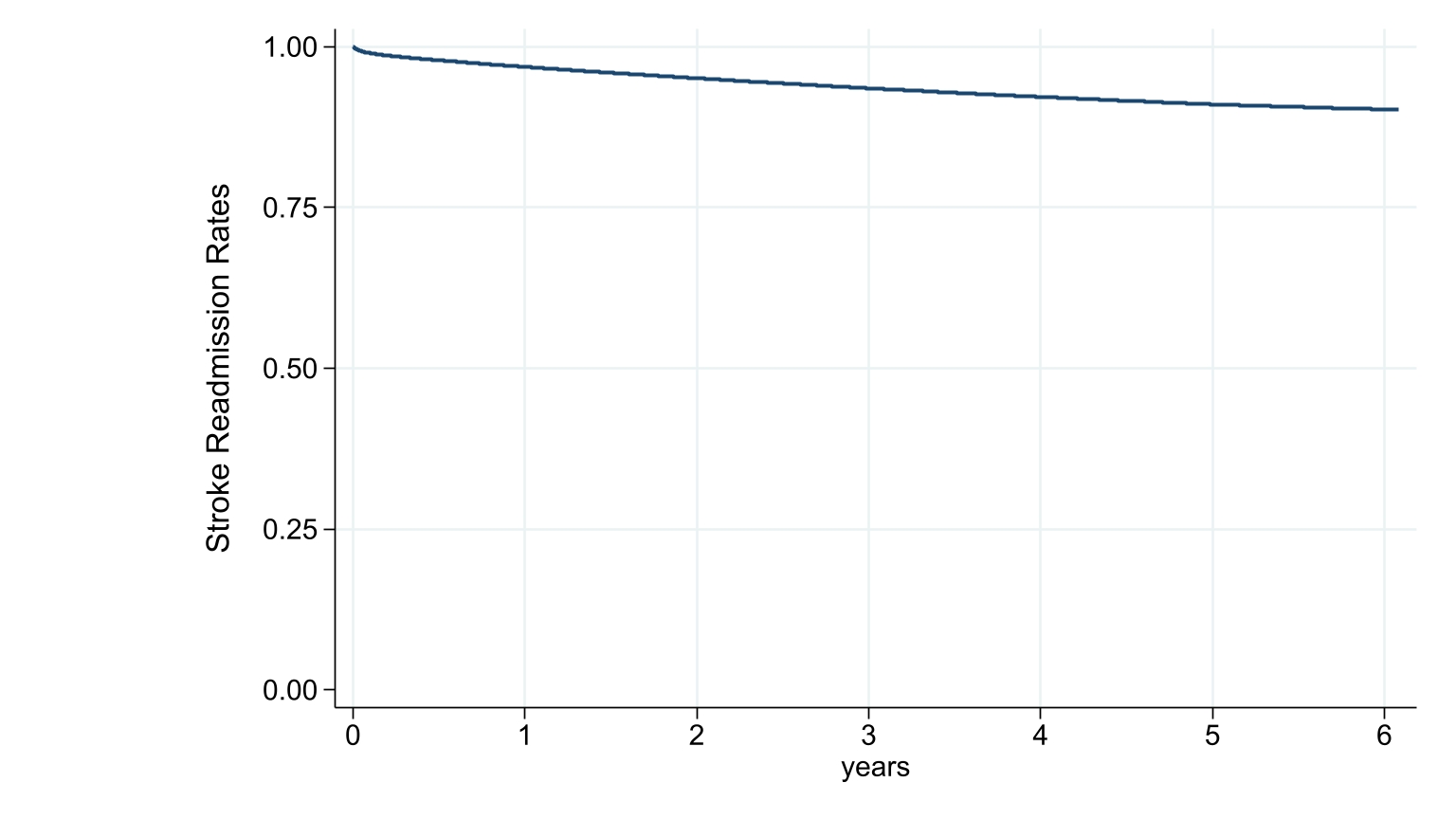
Supplemental Figure S1A: Kaplan Meier Event Curves for All Stroke after TAVR**


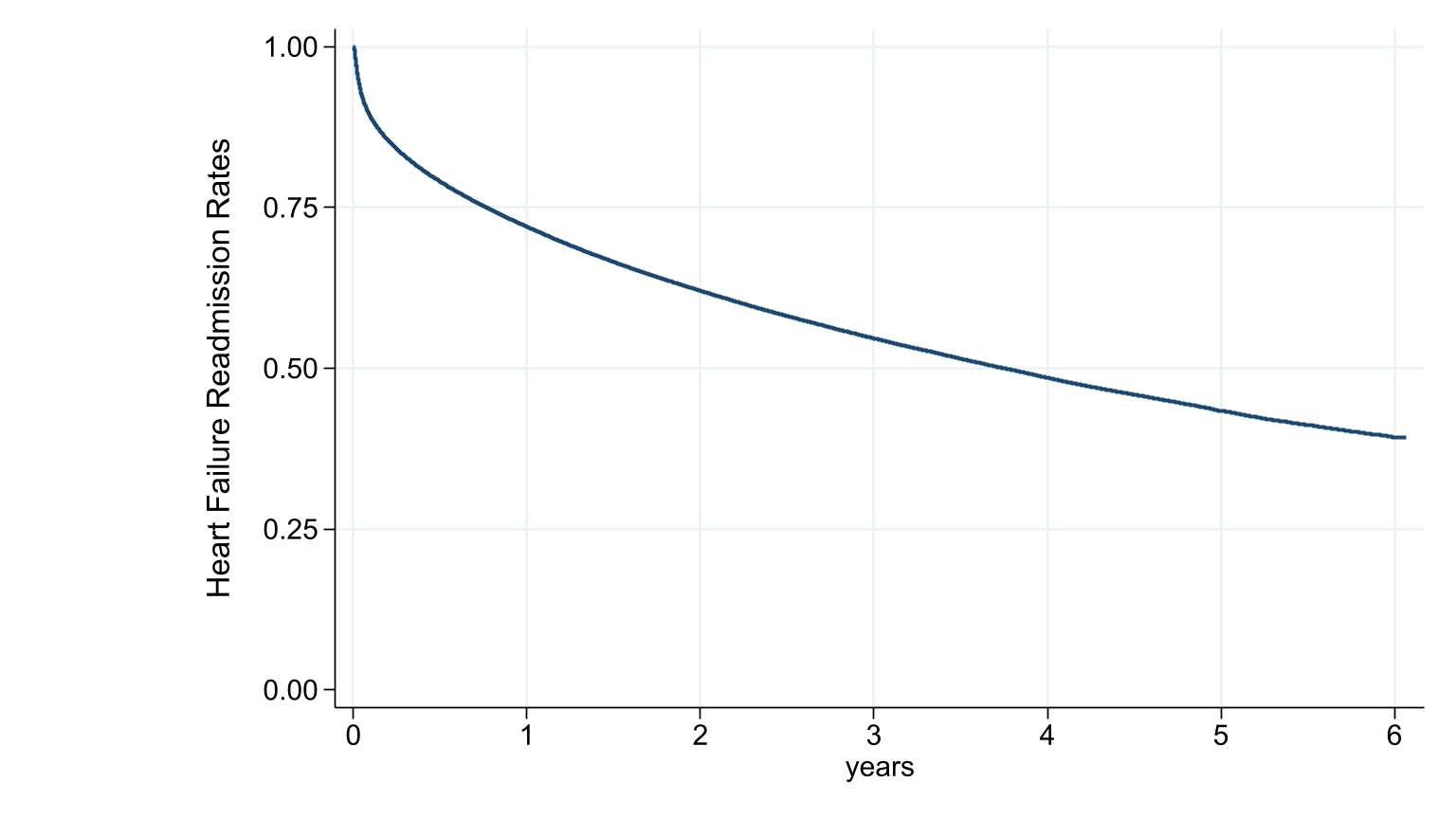
**Supplemental Figure S1B: Kaplan Meier Event Curves for HF Rehospitalization after TAVR**

**
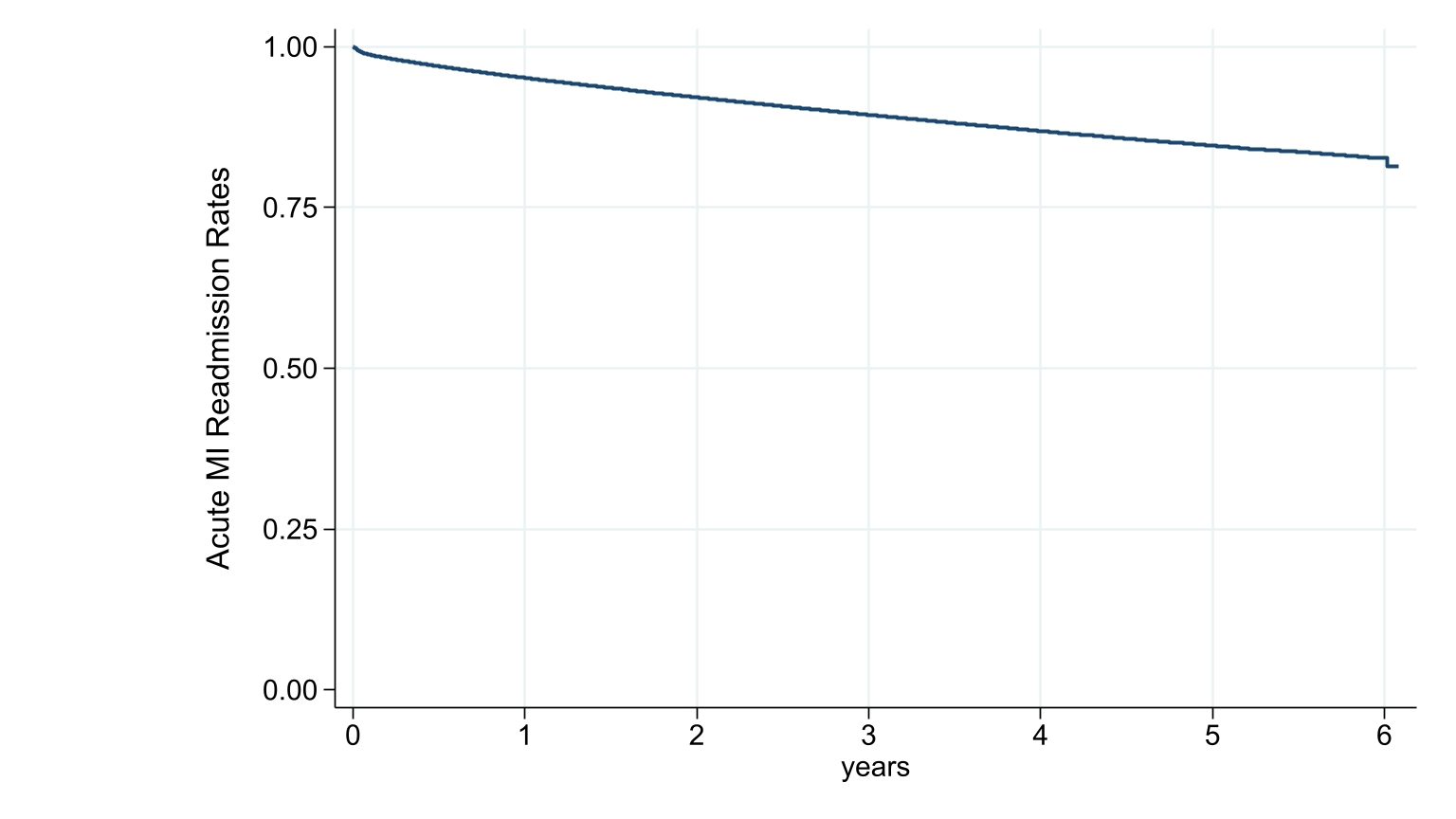
Supplemental Figure S1C: Kaplan Meier Event Curves for Acute Myocardial Infarction after TAVR**
